# Targeting Protein-Protein Interactions with Trimeric Ligands: High Affinity Inhibitors of the MAGUK Protein Family

**DOI:** 10.1371/journal.pone.0117668

**Published:** 2015-02-06

**Authors:** Klaus B. Nissen, Linda M. Haugaard-Kedström, Theis S. Wilbek, Line S. Nielsen, Emma Åberg, Anders S. Kristensen, Anders Bach, Per Jemth, Kristian Strømgaard

**Affiliations:** 1 Department of Drug Design and Pharmacology, University of Copenhagen, Universitetsparken 2, Copenhagen, Denmark; 2 Department of Medical Biochemistry and Microbiology, Uppsala University, Biomedical Centre, Uppsala, Sweden; University of South Florida College of Medicine, UNITED STATES

## Abstract

PDZ domains in general, and those of PSD-95 in particular, are emerging as promising drug targets for diseases such as ischemic stroke. We have previously shown that dimeric ligands that simultaneously target PDZ1 and PDZ2 of PSD-95 are highly potent inhibitors of PSD-95. However, PSD-95 and the related MAGUK proteins contain three consecutive PDZ domains, hence we envisioned that targeting all three PDZ domains simultaneously would lead to more potent and potentially more specific interactions with the MAGUK proteins. Here we describe the design, synthesis and characterization of a series of trimeric ligands targeting all three PDZ domains of PSD-95 and the related MAGUK proteins, PSD-93, SAP-97 and SAP-102. Using our dimeric ligands targeting the PDZ1-2 tandem as starting point, we designed novel trimeric ligands by introducing a PDZ3-binding peptide moiety via a cysteine-derivatized *N*PEG linker. The trimeric ligands generally displayed increased affinities compared to the dimeric ligands in fluorescence polarization binding experiments and optimized trimeric ligands showed low nanomolar inhibition towards the four MAGUK proteins, thus being the most potent inhibitors described. Kinetic experiments using stopped-flow spectrometry showed that the increase in affinity is caused by a decrease in the dissociation rate of the trimeric ligand as compared to the dimeric ligands, likely reflecting the lower probability of simultaneous dissociation of all three PDZ ligands. Thus, we have provided novel inhibitors of the MAGUK proteins with exceptionally high affinity, which can be used to further elucidate the therapeutic potential of these proteins.

## Introduction

Protein-protein interactions (PPIs) are vital for almost all cellular and biochemical processes and have attracted particular attention due to their potential as novel and promising drug targets for the treatment of several disease states.[1,2] The postsynaptic density protein-95 (PSD-95), discs large, zona occludens 1 (PDZ) protein domain family is one of the most wide-spread classes of PPI domains in the human genome, with an estimated 256 PDZ domains in 142 different proteins[3] that facilitates several vital PPIs.[4,5] One PDZ-domain containing protein family, the discs large (dlg) membrane associated guanylate kinase (MAGUK) family, comprises four members, PSD-93, PSD-95, synapse associated protein-97 (SAP-97) and SAP-102. They share the same overall topology of three PDZ domains, one src homology 3 (SH3) domain and one inactive guanylate kinase (GK) domain. The MAGUK proteins are involved in receptor trafficking and scaffolding of primarily post-synaptic signaling complexes through their PDZ domains and have been implicated as potential drug targets for the treatment of neurological diseases such as stroke, chronic pain and Alzheimer’s disease.[4,6–9] In particular, PSD-95 has been shown to function as a scaffolding protein, bridging the *N*-methyl-d-aspartate (NMDA) receptor and neuronal NO synthase (nNOS) via the PDZ1 and PDZ2 domains.[10,11] A 20-mer peptide ligand that disrupts this ternary complex, Tat-NR2B9c (NA-1), has been shown to alleviate neuropathic pain in animal models,[12] reduce ischemic brain damage,[13–17] and has recently successfully passed phase II clinical trials for iatrogenic stoke.[18,19]

The success of NA-1 has led to intense investigations to further explore the function and the development of inhibitors targeting PSD-95. In contrast, much less is known about the function of the other members of the MAGUK family. PSD-93, PSD-95, SAP-97 and SAP-102 all have high sequence similarity (∼80%), similar domain organization and was initially believed to have similar functions,[20] supported by *in vitro* binding studies showing similar binding specificities.[21] Although the MAGUK proteins have overlapping functions, exemplified by the compensation by PSD-93 in PSD-95 knockout animals,[22] notable functional differences of the MAGUK family of proteins exist, such as different affinities of SAP-102 and PSD-95 for particular subunits of the NMDA receptor.[23–26] However, the exact functions and the disease relevance of PSD-93, SAP-97 and SAP-102 are still poorly understood and genetic knock-out studies are difficult to interpret due to functional compensation of these proteins.[6,8,27] Moreover, due to the high sequence similarity of the proteins, PSD-95 inhibitors are likely nonselective among the MAGUK proteins.[28,29]

It was recently shown that PSD-95, SAP-97 and SAP-102 are organized into two distinct functional supramodules, consisting of PDZ1-2 and PDZ3-SH3-GK domains, respectively. A highly flexible peptide linker between PDZ2 and PDZ3 connects the two supramodules.[30,31] This linker is the least similar region of the MAGUK family and the linker length differs from 61 to 141 residues within the family.[32] However, the linker appears not to be extended based on intramodular distance estimates in PSD-95, SAP-97 and SAP-102 as measured by single molecule fluorescence energy transfer.[31] The functional consequences of this difference in linker length and sequence on binding to multi-domain membrane receptors or multivalent inhibitors are currently unknown.

**Fig 1:**
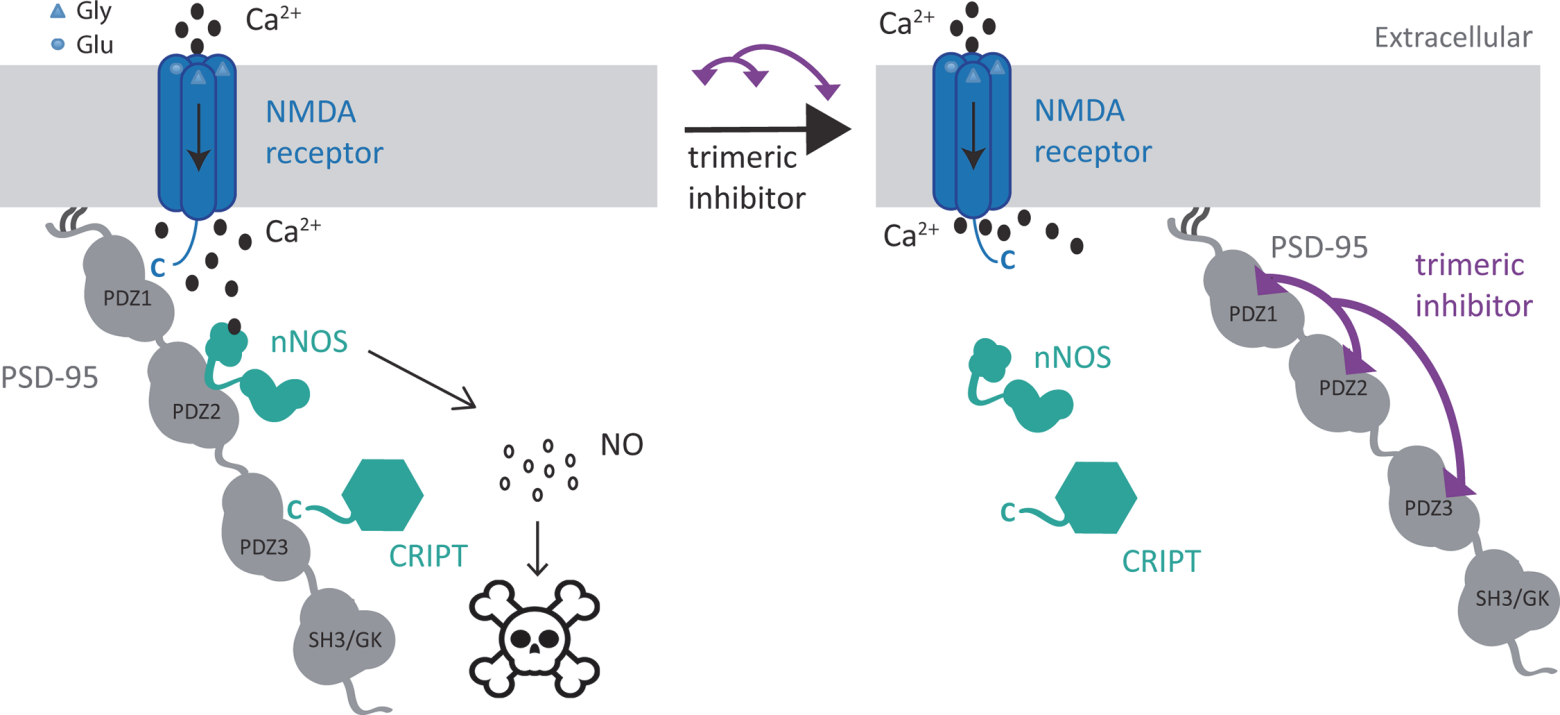
Illustration of the proposed mode of action against excitotoxicity by inhibition of PSD-95. During ischemia and stroke a large amount of glutamate is released, which activates the NMDA receptors. Upon NMDA receptors activation Ca^2+^ influx occurs, which stimulate PSD-95 regulated activation of nNOS and the production of NO. By blocking the NMDA receptor/nNOS/PSD-95 complex with a trimeric peptide inhibitor targeting PSD-95, the connection between NMDA receptor activation and toxic NO production is blocked, whereby neuroprotection against excitotoxicity is achieved.

We have previously developed high affinity dimeric ligands targeting PDZ1-2 of PSD-95, which have 1000-fold improved affinity over monomeric peptides ligands, improved plasma stability and have shown great promise in a mouse model of stroke.[33,34] Here, we explore the concept of multivalent PDZ ligands further and describe the design, synthesis and evaluation of trimeric ligands that simultaneously target all three PDZ domains of the MAGUK proteins (Fig. 1). It is tempting to speculate that ligands spanning the PDZ2 and PDZ3 domains could be employed as elements that stabilize this region and thereby allow high-resolution structural studies. Furthermore, these ligands could potentially be used as model compounds to study the functional consequences of targeting both supramodules of the MAGUK proteins simultaneously. The study resulted in ligands with very high affinity towards PDZ1-2-3 and full length proteins and selectivity over single PDZ domains.

## Materials and Methods

### Chemistry

Amino acids, preloaded Wang resins and 2-chlorotrityl chloride resin, *O*-(benzotriazol-1-yl)-*N*,*N*,*N*′,*N*′-tetramethyluronium hexafluorophosphate (HBTU) and *O*-(7-azabenzotriazol-1-yl)-*N*,*N*,*N*′,*N*′-tetramethyluronium hexafluorophosphate (HATU) were purchased from IRIS Biotech (Marktredwitz, Germany). Cy5 maleimide was purchased from GE lifescience and the PEG building blocks were obtained from Biomatrik Inc (Jiaxing, China) with one exception, the Malonyl-PEG(28)-N-hydroxysuccinimide (Mal-PEG(28)-NHS), which was obtained from Polypure (Oslo, Norway). The remaining reagents were obtained from Sigma-Aldrich (St. Louis, MO, USA). Small-scale preparative reverse phase high-performance liquid chromatography (RP-HPLC) was performed on an Agilent 1200 (Agilent Technologies, Santa Clara, CA, USA) system using a C18 reverse phase column (Zorbax 300 SB-C18, 21.2 mm × 250 mm) with a linear gradient of the binary solvent system of H_2_O/acetonitrile (ACN)/trifluoroacetic acid (TFA) (A, 95/5/0.1, and B, 5/95/0.1) with a flow rate of 20 mL/min. Large-scale preparative C18 RP-HPLC was performed on a Biotage Flashmaster (Biotage, Uppsala, Sweden) equipped with a Redi-sep Rf Gold 150 gram HP C18 coloumn using the same liquid system as for the Agilent system and a flow rate as recommended by the Flashmaster software. Analytical HPLC was performed on an Agilent 1100 system with a C18 reverse phase column (Zorbax 300 SB-C18 column, 4.6 mm × 150 mm), flow rate of 1 mL/min, and a 1%/min linear gradient of the binary solvent system of H_2_O/ACN/TFA (A, 95/5/0.1, and B, 5/95/0.1). Mass spectra were obtained with an Agilent 6410 Triple Quadrupole Mass Spectrometer instrument using electron spray ionization (ESI) coupled to an Agilent 1200 HPLC system (ESI-LC/MS) with a C18 reverse phase column (Zorbax Eclipse XBD-C18, 4.6 mm × 50 mm), autosampler and diode array detector using a 1%/min linear gradient of the binary solvent system of H_2_O/ACN/formic acid (A, 95/5/0.1, and B, 5/95/0.086) with a flow rate of 1 mL/min. During ESI-LC/MS analysis, evaporative light scattering (ELS) traces were obtained with a Sedere Sedex 85 Light Scattering Detector. Matrix-Assisted Laser Desorption Ionization-Time of Flight (MALDI-TOF) mass spectra were obtained on a Bruker Microflex LT system. A 10 mg/mL solution of α-Cyano-4-hydroxycinnamic acid was used as matrix. Nuclear Magnetic Resonance (NMR) experiments were conducted on a Bruker 400 MHZ NMR with cryogenic cooling. Chemical shifts (*δ*) are reported in parts per million (ppm) with reference to tetramethylsilane (TMS) as internal standard. NMR experiments were carried out in CD_3_OD. The following abbreviations are used for the proton spectra multiplicities: s, singlet; t, triplet; m, multiplet. Coupling constants (*J*) are reported in Hertz (Hz). TLC analysis was performed on silica gel F_254_ (Merck) and detection was carried out by examination under UV light and staining with potassium permanganate. Flash column chromatography was performed on silica gel with solvent of HPLC grade.

### Peptide synthesis

Peptide sequences were synthesized by Fmoc-based solid phase peptide chemistry using preloaded Fmoc-Val-Wang-resin or Val-2CT-resin (0.6–0.7 mmol/g, 100–200 mesh), HBTU/ diisopropylethylamine (DIPEA) for couplings, and dry DMF as solvent. Each coupling was carried out for 45 min with resin/Fmoc-amino acid/HBTU/DIPEA (1:4:4:4), and was qualitatively evaluated by the ninhydrin test. Fmoc-deprotection was carried out in 20% piperidine in DMF for 5 min, followed by DMF wash and a second piperidine/DMF treatment for 5 min.

### Purification and characterization

Compounds were obtained as TFA salts by treating the resin-bound products with TFA/triisopropylsilane (TiPS)/H_2_O (90/5/5) for 2 h followed by filtration, evaporation *in vacuo*, precipitation with cold ether, lyophilization, and purification with preparative RP-HPLC. All final compounds were characterized for purity (>95%) (analytical RP-HPLC) and molecular mass by ESI-LC/MS and MALDI-TOF MS.

### Synthesis of side-chain protected CRIPT-06 (1) and CRIPT-2Ala (9)

To obtain the fully side-chain protected peptides (**1** and **9**), the resin was treated with 20% hexafluoroisopropanol (HFIP) in DCM (2 x15 mL, 2 x5min). The combined HFIP/DCM fractions were filtered through silica, evaporated and co-evaporated twice from DCM and lyophilized. The resulting peptide fragments were highly pure (>95%) as judged from LC-MS and used without further purification. The side-chain protected CRIPT-2Ala (**9**) was synthesized in the same manner, with the exception that the peptide sequence YKQASA was used.

### Synthesis of malonylpropionyl-PEG(n)-CRIPT conjugates (2–8)

To generate the MAL-PEG-CRIPT conjugates (**2–7**), 0.1 mmol (1 eq.) of MAL-PEG-OPfp or MAL-PEG-NHS, 27.2 mg (0.2 mmol, 2 eq.) hydroxyazabenzotriazol (HOAt) and 2 eq. DIPEA in 5 mL DCM were added to 0.1 mmol (1 eq.) side-chain protected CRIPT-06 (**1**) in 1 mL DMSO. The solution was stirred vigorously and the reaction was followed by LC-MS. The reaction was complete after one hour, after which the solution was evaporated to dryness. The white residue was then treated with TFA/TiPS/H_2_O (90/5/5, 10 mL) for 2 hours. The TFA was removed by evaporation and the residue was washed with ether and lyophilized. The crude product was purified by small-scale preparative RP-HPLC. The MAL-PEG(12)-CRIPT-2Ala (**8)** was synthesized in the same manner, by a reaction between MAL-PEG(12)-OPfp and side-chain protected CRIPT-2Ala (**9**). The characterization of the conjugates is described in S6 Table.

### Synthesis of Cys-*N*-dimer (11)

The *N*-dimer **10** was synthesized as previously described.[34] Cys was coupled to the nitrogen in **10** by 2 consecutive couplings. For both couplings Fmoc-Cys(Trt)-OH (4 eq) was activated with *O*-(7-azabenzotriazol-1-yl)-*N*,*N*,*N*′,*N*′-tetramethyluronium hexafluorophosphate (HATU) in DMF (4 eq, 0.5M) and collidine (8 eq), before addition to (**10**). The coupling was repeated after 2 h and followed by a DMF wash. The Fmoc group was removed by treating the resin with 20% piperidine in DMF for 5 min, followed by a second treatment. The compound was cleaved from the resin by treatment with TFA/TiPS/H_2_O (90/5/5) and purified by large-scale C18 RP-HPLC.

### Synthesis of trimeric ligands (12–17 and 21)

Equimolar amounts of MAL-PEG-CRIPT (**2**–**8**) and the Cys-*N*-dimer (**11**) were diluted in 2 mL 40% ACN in H_2_O. After dissolution, 2 mL phosphate buffered saline, pH 7.2 (PBS, 10 mM HPO_4_/H_2_PO_4_, 150 mM NaCl) was added and the reaction was stirred for 1 h. After the reaction had completed, excess thiol was quenched with 1M mercaptoethanol and the trimer was isolated by purification on a HiLoad 16/600 Superdex 30 coloumn using an Äkta FPLC system (GE Life Sciences, Uppsala, Sweden) at a flow of 0.5 ml/min. The characterization of the tridentate ligands is described in S5 Table.

### Synthesis of Cy5-Cys-*N*-dimer (18)

**11** (2.048 mg, 1.32 μmol) was solubilized in 10×TBS buffer (1.4 mL; 1.5 M NaCl, 100 mM Tris, pH 7.4, degassed). Cy5-maleimide (1.358 mg, 1.74 μmol) was solubilized in DMSO (40 μL) and mixed with the solution of **11**. The reaction was incubated overnight at room temperature and the final product was purified by C18 RP-HPLC. Yield: 2.3 mg (0.985 μmol, 75%). m/z (ESI): 1167.8 [M+2H]^2+^ (100); 779.1 [M+3H]^3+^ (14). Purity >95% (ELSD, UV).

### Synthesis of dimeric ligand UCCB01–125 (20)

**20** was synthesized as previously described.[33,34]

### Synthesis of monomeric ligand dansyl-1

Dansyl was coupled to ligand **1** on resin by adding 8 eq dansyl-Cl and 6 eq DIPEA in DCM. The reaction was allowed to proceed for 1 h in room temperature and was monitored by LC-MS. The peptide was cleaved from the resin by treatment with TFA/TIPS/H_2_O (95/2.5/2.5) for 2 h. Purification was performed according to the general procedure, as stated above.

### Fluorescence polarization (FP) assay

The monomeric FP assay was conducted as previously described except that the proteins were diluted in 50 mM TRIS, 100 mM NaCl, 1 mM ethylenediaminetetraacetate (EDTA), 1 mM DTT.[34] The monomeric probe was only used when monomeric binding to PSD95 PDZ1-2 and PSD95 PDZ1-2-3 was evaluated, whereas **18** was used as probe for evaluating the binding of the dimeric and trimeric ligands. To generate a saturation curve and hence determine *K*_d_, a probe concentration of 0.5 nM was used together with varying protein concentrations (0–256 nM). The inhibition experiments were based on a 12–14 point determination with 0.5 nM probe, the above-mentioned buffer, a fixed protein concentration and varying peptide concentrations (0–8200 nM). In all of the experiments a blank (buffer) and a reference sample (0.5 nM probe in buffer) were used. A total of 30 μl was pipetted into the wells of a 384 flat black bottom well plate. The FP was measured on a Tecan Safire 2 microplate reader (Tecan Group LTD., Männedorf, Germany). The absorption/emission wavelengths were set to 635/670 nm, corresponding to the Cy5 spectra. The obtained IC_50_ values were converted to *K*_i_ values using the equations described by Nikolovska-Coleska et al.[35] The affinity of **15** for SAP-97 PDZ1-2-3 and SAP-102 PDZ1-2-3 could not be calculated due to the properties of equation used to calculate the K_i_-value. Consequently, the Ki-value will become negative when the difference between the IC_50_-value and the K_d_ is too great.[35,36] The IC_50_ values are shown in S3 Table.

### Stopped-flow spectrometry

Kinetic experiments using stopped-flow were carried out under the conditions used previously,[37,38] namely in 50 mM potassium phosphate, pH 7.5 at 10°C. The change in fluorescence upon binding was monitored with a 330 nm band-pass filter following excitation at 280 nm. For binding experiments to determine *k*_on_, FL PSD-95 (2 μM, final concentration after mixing), with either the I100W or the I195W mutation, was mixed with the tridentate ligand **15** (1–12 μM, final concentration). Binding experiments were also performed between FL PSD-95 (wild-type, I100W and I195W, respectively) and a dansyl-labeled CRIPT-06. Experimental traces were fitted to a single exponential function and the *k*_obs_ values thus obtained were plotted versus the concentration of **15** and fitted to an equation for the second order association of two molecules[37,39] to obtain *k*_on_^app^. Regarding the fluorophores and the observed changes in fluorescence, several events contribute to the observed signal. Upon excitation of Trp at 280 nm, both the dansyl and the 5-FAM fluorophores quench the observed Trp fluorescence on binding to FL PSD-95 I100W and I195W, respectively. This could be either through FRET or by absorbing excitation light. Both fluorophores are also themselves excited at 280 nm (less) and 330 nm (more) and their intrinsic fluorescence may change on binding to the proteins. For displacement experiments to determine *k*_off_^app^, 2 μM of FL PSD-95 (I100W and I195W in separate experiments) was pre-incubated with 2–3 μM of **15** and mixed 50:50 in the stopped-flow with either 5-FAM-labeled compound **20** (1–20 μM) or dansyl-labeled CRIPT-06 (**2**) (5–250 μM). The kinetic phase resulting from displacement of **15** with the 5-FAM-**20** compound decreased with increasing [5-FAM-**20**] as expected.[37,38] In the binding and displacement experiments with tridentate inhibitor **15**, excitation was at 280 nm and the emission was monitored using a 330 nm band-pass filter.

A similar set of experiments, as performed for FL PSD-95 and tridentate inhibitor **15,** was performed for FL PSD-95 I100W with the dimeric ligand **20**, to obtain *k*_on_^app^ and *k*_off_^app^, for comparison with the tridentate ligand. As previously observed,[40] binding was biphasic and the fast linear phase was used to estimate *k*_on_^app^. In the set of experiments in which dansyl-**1** was used to displace the complex between FL PSD-95 (I100W and I195W) biexponential traces were observed when excitation was at 280 nm and emission monitored with the 330 nm. One phase displayed the typical decrease with increasing concentration of competitor (stabilizing at around 0.5 s^-1^ at high [dansyl-**1**]), while the other phase increased hyperbolically to a value of around 2.5 s^-1^. We speculate that the observed phases result from partial dissociation of **15** at PDZ3 of FL PSD-95, possibly in combination with binding of dansyl-**1** to all three PDZ domains at the high concentrations used. (A slow phase observed for direct binding of dansyl-**1** to FL PSD-95 could reflect a conformational change and may also contribute to the complex dissociation kinetics observed here.) The displacement experiment with dansyl-**1** was conducted both with excitation at 280 nm/330 nm band-pass filter (effectively monitoring the engineered Trp), but also using excitation at 330 nm (absorbance maximum for dansyl) and emission monitored at >420 nm. The latter setup resulted in kinetic traces, which were reasonably well-described by a single exponential function (reported in S1C Fig). This observed rate constant might be the average of the two *k*_obs_ values observed upon excitation at 280 nm/emission at 330 nm. In any case, the apparent dissociation rate constant of the FL PSD-95/tridentate ligand **15** complex is close to that observed for PDZ3/**1** as discussed in the main text. Binding of dansyl-**1** to FL PSD-95 (wild-type, I100W and I195W, S1A–B Fig) was monitored by excitation at 330 nm/emission at >420 nm using a cut-off filter.

## Results and Discussion

### Design and synthesis

**Fig 2:**
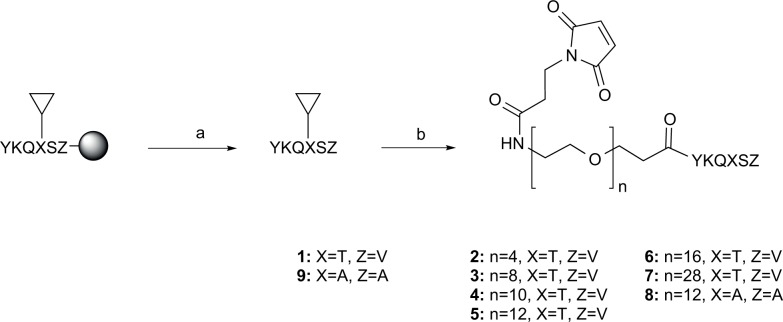
Synthesis of building blocks 1−9. Reaction conditions: (a) HFIP, DCM (2 x 5 min); (b) malonylpropionyl-PEG(n)-O-PfP or malonylpropionyl-PEG(n)-O-NHS, DIPEA, HOAt, DMF, DCM, then TFA,TiPS, H2O (90:5:5). ▽ = fully side-chain protected peptide. Single letters designate amino acids except for N (nitrogen), O (oxygen) and H (hydrogen).

In our design of trimeric ligands targeting all three PDZ domains (PDZ1-2-3) of the MAGUK proteins, we took advantage of our work on dimeric ligands targeting the tandem PDZ1-2 domain of PSD-95. Since PDZ1-2 is part of the same supramodule and the PDZ3 domain is part of another, we envisioned that our optimized dimeric ligands could be used as templates and by adding a third binding module targeting PDZ3 would generate trimeric ligands that simultaneously bind PDZ1-2-3. This could be achieved by exploiting the polyethylene glycol (PEG) based linker containing a secondary amine, the *N*PEG linker, which was used for dimeric ligands with cell-penetrating peptides.[34] Subsequently, the secondary amine in the *N*PEG linker could be derivatized with another linker containing a PDZ3 binding peptide. We selected the C-terminal sequence of the cysteine-rich interaction partner of PDZ3 (CRIPT)[41] as the PDZ3 binding ligand. We have previously shown that a pentapeptide ligand is sufficient to provide wild type affinity for PDZ1 and PDZ2 of PSD-95,[42] hence we performed a truncation study of the CRIPT peptide ligand, to examine the minimal sequence providing wild-type affinity for PDZ3 of PSD-95. In agreement with a previous study,[43] we found that a hexapeptide ligand, YKQTSV, was required for wild type affinity at PDZ3 (S1 Table). We envisioned that a convenient way to attach the linker-PDZ3 ligand building block to the *N*PEG, would be to first derivatize the *N*PEG secondary amine with a cysteine and subsequently react the cysteine with malonyl containing linker-PDZ3 ligand building blocks in a cysteine-maleimide reaction to generate trimeric ligands. Since we did not know the optimal linker length, we generated linker-PDZ3 ligand building blocks by reaction of the CRIPT hexapeptide (YKQTSV, **1**) with malonylpropionyl-PEG-COOH having different PEG linker length (Fig. 2).

The linker-PDZ3 ligand building blocks were synthesized by fragment coupling in solution: Fully side-chain protected peptide **1** was obtained by standard Fmoc-based solid-phase peptides synthesis and cleavage from the resin with 20% 1,1,1,3,3,3-hexafluoroisopropanol (HFIP) in dichloromethane, as previously described.[44] The free N-terminal was coupled with pre-activated pentaflourophenyl (Pfp) or *N*-hydroxysuccinimide (NHS) esters of malonylpropionyl-PEG-COOH in solution, followed by deprotection and purification by RP-HPLC to provide building blocks **2–7** with six different PEG linker linkers ranging from PEG4 to PEG28 (Fig. 2). In addition, a non-binding linker-PDZ3 building block, malonylpropionyl-PEG(12)-CRIPT-2Ala (**8**), derived from the double Ala mutated CRIPT peptide, YKQASA (**9**), was prepared in the same manner (Fig. 2).

**Fig 3:**
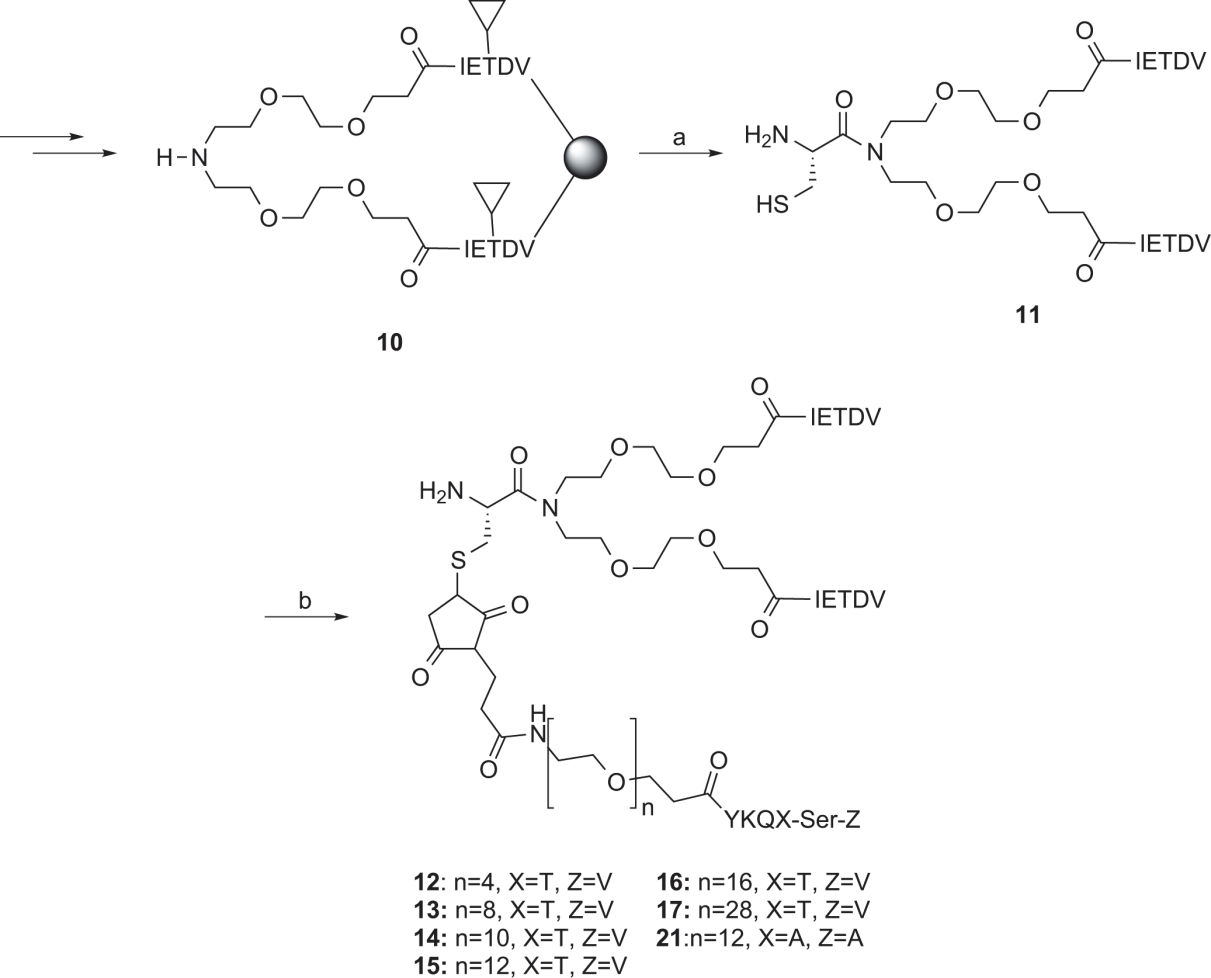
Synthesis of building blocks 10–17 and 21. Reaction conditions: (a) 1. Fmoc-Cys(Trt)-OH, HATU, collidine, DMF (2x45 min), 2. 20% Piperidine in DMF, 3. TFA,TIPS, EDT, H_2_O (90, 2.5, 2.5, 5); (b) **3−8** or **10**, PBS pH 7.2, ACN (75:25) ▼ = fully side-chain protected peptide. Single letters designate amino acids except N (nitrogen), O (oxygen), H (hydrogen) and S (sulfur).

For the synthesis of the dimeric ligand template we prepared the *N*PEG IETDV dimer (**10**) as previously described.[34] Subsequently, the Cys-*N*PEG dimer (**11**) was obtained by coupling of Fmoc-Cys(Trt)-OH to the secondary amino group in the *N*PEG linker by repeated peptide couplings followed by Fmoc deprotection, cleavage from the resin and concomitant global deprotection by TFA and purification by preparative RP-HPLC, in 43% yield (Fig. 3). Finally, the trimeric ligands **12–17**, were readily obtained by reaction of the linker-PDZ3 building blocks **2–8** with **11** in water/acetonitrile at pH 7.2, followed by purification by size-exclusion chromatography (Fig. 3).

For the subsequent evaluation in a fluorescence polarization (FP) assay, we have previously used a dimeric fluorescence probe using a 5-carboxyfluorescein fluorophore for determination of the high affinity dimeric ligands.[34] Here, we used a slightly different probe based on the Cy5 fluorophore, **18**, which has an improved quantum yield. The fluorescent probe was conveniently synthesized using **11** as an intermediate, and reacting commercially available Cy5 containing a maleimide moiety at pH 7.4 followed by RP-HPLC purification, providing **18** (See supporting information).

### Proof of concept for trimeric ligands

First, we wanted to examine the feasibility of the trimeric ligand concept i.e., that three peptide ligands could in fact bind the three PDZ domains (PDZ1-2-3) of PSD-95 simultaneously. Therefore, we first derivatized the Cys-*N*PEG dimer with the linker-PDZ3 ligand moiety comprising a long PEG28-linker, leading to trimeric ligand **17**. We then expressed PDZ1-2-3 domains from the four human MAGUKs, PSD-93, PSD-95, SAP-97 and SAP-102, as His-tagged fusion proteins in *E*. *coli* and purified them using immobilized metal ion-affinity chromatography (IMAC) followed by ion exchange chromatography. Finally, we employed full length (FL) PSD-95, which was generously donated by Professor Mingjie Zhang, Hong Kong University of Science and Technology.[45]

**Fig 4:**
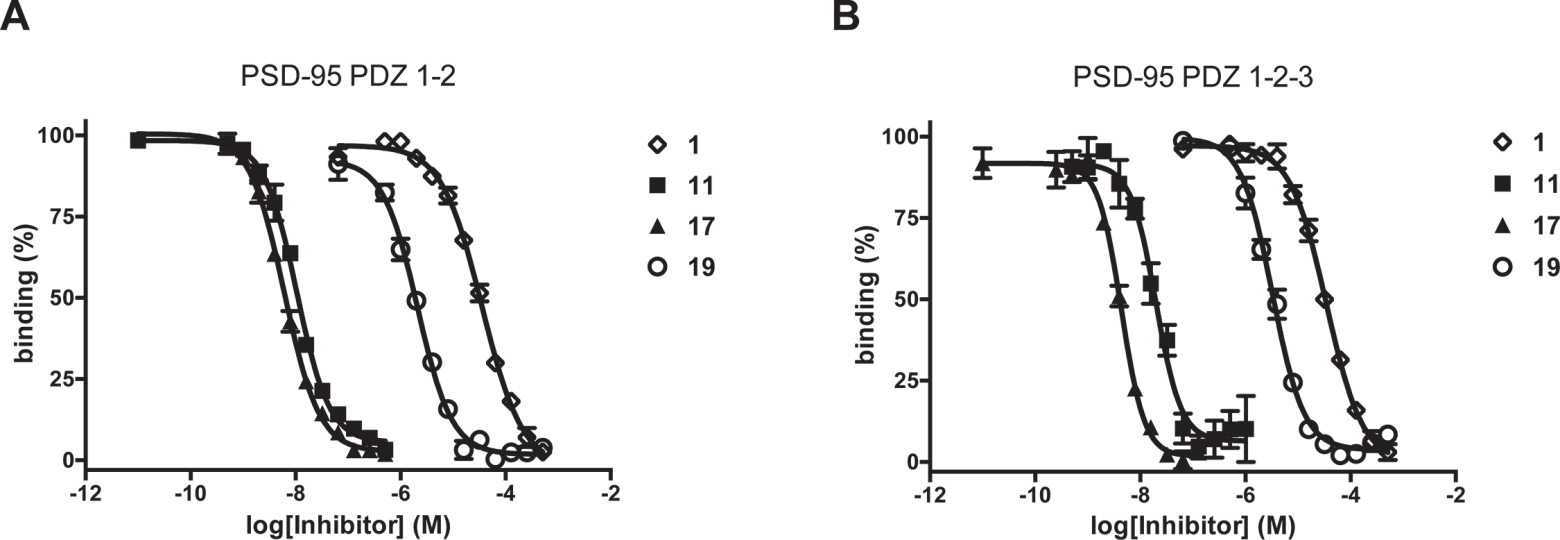
FP binding curves of 1, 11, 17 and 19 for PSD-95 PDZ1-2 (A) and PSD-95 PDZ1-2-3 (B). Data plotted as mean ± SEM, n≥3.

To investigate whether the trimeric ligand **17** indeed bound to PDZ 1-2-3 of PSD-95 as a trimeric ligand, we examined the affinity of **17** together with the dimeric ligand **11** to both PDZ1-2 and PDZ1-2-3 constructs of PSD-95 using an FP assay employing the dimeric fluorescent probe **18**. In addition, the affinities of the individual monovalent peptides in the trimeric ligand molecule were examined as controls; these were YKQTSV (**1**) targeting PDZ3 and IETDV (**19**) targeting PDZ1 and PDZ2. When tested towards PSD-95 PDZ1-2 the trimeric ligand **17** and dimeric ligand **11** showed comparable affinities, as anticipated (Fig. 4). However when measuring the affinity to PSD-95 PDZ1-2-3, it was observed that trimeric ligand **17** had an increased affinity relative to **11** (Fig. 4), as **17** binds with an approximately 3-fold higher affinity than **11** (Fig. 4). This demonstrates that adding a third peptide binding moiety to the dimeric ligand enhances affinity towards PDZ1-2-3 and thus indicates that trimeric ligand **17** engages in a trivalent binding-mechanism involving all three PDZ domains of PDZ-1-2-3 of PSD-95. Moreover, monomeric peptide ligands **1** and **19** were also tested for affinity towards PDZ1-2 and PDZ1-2-3, showing >1000-fold lower affinities compared to trimeric ligand **17** (Fig. 4), as shown previously for the dimeric ligands.[33] The affinity of **17** and the monomeric peptides **1** and 1**9** towards the individual PDZ domains (PDZ1, PDZ2 and PDZ3 of PSD-95) were also measured using monomeric fluorescent peptide probes (S2 Table), which showed that the trimeric ligand **17** had a similar affinity as monomeric ligands **1** and **19** when measured towards single PDZ domains (S2 Table). Thus, the trimeric ligand **17** binds with very high affinity to PDZ1-2-3 of PSD-95 and is more potent than dimeric ligand **11,** supporting that as set out in the design of trimeric ligands each of the three peptide binding moieties of **17** bind to the corresponding PDZ domains of PSD-95.

### Optimization of trimeric ligands

With a proof of concept from our trimeric ligand **17**, which contained a very long PEG linker between the dimeric ligand and the PDZ3 ligand binding peptide, we wanted to investigate if the affinity of trimeric ligands could be optimized by adjusting this linker length. Thus, we designed a series of corresponding trimeric ligands with variation of the PEG linker length to the PDZ3 binding peptide ligand. Since **17** contained a linker with a maximal PEG28 unit, we selected a series of shorter PEG linkers that could provide information of the optimal linker lengths. Trimeric ligands **12–16** containing PEG4, PEG8, PEG10, PEG12 and PEG16, respectively, were synthesized and the affinities towards PDZ1-2-3 of PSDS-95 were determined in the FP assay. For comparison, we measured the affinity of our most studied, high-affinity dimeric ligand UCCB01-125[33] (**20**) toward the same protein. Gratifyingly, all the trimeric ligands displayed higher affinity towards PSD-95 PDZ1-2-3 compared to the dimeric ligand **20** with *K*_i_ values in the low nanomolar range, from 3.3 to 16 nM (Table 1), and the dimeric ligand **20** had an affinity in the same range as previously measured toward PSD-95 PDZ1-2 (*K*_i_ = 26.5 ± 1.5 nM, Table 1).[33] Interestingly, we found a clear optimal linker length of the trimeric ligands, as compound **15** containing a PEG12 linker had the highest affinity and showed an 8-fold increase in affinity towards PSD-95 PDZ1-2-3 relative to the dimeric control compound **20** (Table 1).

**Table 1:** Affinity of trimeric ligands (12–17 and 21) and dimeric ligand 20 toward the PDZ1-2-3 tandems of PSD-93, PSD-95, SAP-97 and SAP-102 and FL PSD-95 as determined by FP*a*.

**Compound**	**PSD-93 PDZ1-2-3**	**PSD-95 PDZ1-2-3**	**SAP-97 PDZ1-2-3**	**SAP-102 PDZ1-2-3**	**PSD-95 FL**
	*K*_*i*_ (nM)		*K*_*i*_ (nM)		*K*_*i*_ (nM)
**12**	11.5 ± 1.3	12.5 ± 1.8	5.8 ± 1.3	7.9 ± 2.0	10.9 ± 1.0
**13**	10.9 ± 2.1	7.9 ± 2.0	7.0 ± 1.3	9.9 ± 1.8	10.3 ± 1.0
**14**	8.3 ± 3.0	15.9 ± 3.0	5.9 ± 2.6	8.8 ± 3.9	15.3 ± 3.1
**15**	3.0 ± 0.4	3.3 ± 0.7	#	#	2.2 ± 0.6
**16**	9.3 ± 1.9	12.5 ± 1.3	7.1 ± 2.0	9.2 ± 2.4	13.7 ± 1.4
**17**	4.9 ± 0.6	9.7 ± 1.0	5.3 ± 0.4	11.1 ± 1.3	32. 3 ± 3.5
**21**	15.0 ± 1.8	38.6 ± 3.4	10.1 ± 1.7	15.4 ± 3.1	37.3 ± 6.0
**20**	15.1 ± 0.8	26.5 ± 1.5	9.2 ± 0.9	32.8 ± 5.5	27.2 ± 2.2

^*a*^Data shown as mean ± SEM in nM, n≥3. *K*_i_ values calculated according to Nikolovska-Coleska et al., 2004[35]. # = calculated *K*_i_ is negative, due to limitations of the FP assay, see also S3 Table.[36],[35]

The four human MAGUK proteins (PSD-93, PSD-95, SAP-97 and SAP-102) show a very high degree of sequence similarity, and their PDZ domains bind similar peptide ligands, thus dimeric ligands would not be expected to be able to discriminate among these proteins. This is also what we find, when determining the affinity of dimeric ligand **20** towards PSD-95 PDZ1-2-3, as well as PDZ1-2-3 from the other three MAGUK proteins, PSD-93, SAP-97 and SAP-102, showing *K*_i_ values of 26.5 ± 1.5, 15.1 ± 0.8, 9.2 ± 0.9 and 33 ± 6 nM, respectively (Table 1). However, one of the primary differences among these MAGUK proteins lies in the linker between PDZ2 and PDZ3 and thus it could be envisioned that trimeric ligands are more likely to display selectivity compared to their dimeric counterparts. We therefore tested the series of trimeric ligands (**12–17)** towards the PDZ1-2-3 proteins from PSD-93, SAP-97 and SAP-102. All trimeric ligands showed increased affinity to all MAGUK proteins relative to dimeric ligands and trimeric ligand **15**, with a PEG12 linker to the PDZ3 binding ligand, had the highest affinity to all PDZ1-2-3 proteins. However, the affinities among the different MAGUK proteins did not differ significantly (Table 1), suggesting that the differences in the flexible peptide region between the PDZ1-2 and PDZ3-SH3-GK supramodules of the MAGUKs does not lead to differences when it comes to recognizing and interacting with trivalent ligands. The length of the PEG12 linker can be estimated to approximately 5.3 nm, which agrees well to the estimated distance between the PDZ1-2 and PDZ3-SH3-GK supramodules of 4.1–7.2 nm.[31]

In an attempt to further verify that the increased affinity of trimeric ligand **13** was caused by a trivalent binding to PDZ1-2-3, we prepared a trimeric ligand (**21**) with the same structure as **13**, but where the PDZ3 binding sequence (YKQTSV) was replaced with a double Ala mutant sequence (YKQASA). It is well-established that Ala mutations in position 0 and -2 of PDZ peptide ligands abolishes binding and this was also confirmed for the monomeric CRIPT sequence and its double Ala mutant by FP experiments (S1 Table).[33] The affinity of the trimeric ligand **21** with a non-binding PDZ3 ligand would be expected to have an affinity towards PDZ1-2-3 similar to that of the dimeric ligand **20** and our examination of the four PDZ1-2-3 proteins confirmed this: The affinity of **21** was in the same range as **20** and markedly reduced compared to **15** (Table 1), in agreement with a trivalent binding interaction of **15**.

### Trivalent binding to full length PSD-95

Recent studies have shown that the PDZ3 domain associates with the SH3-GK domains in a supramodule in the MAGUK proteins.[30,31,46] So far, we have examined the affinity towards the PDZ1-2-3 constructs of the MAGUK proteins, however it is clearly relevant to investigate whether the association of PDZ3 with SH3 and GK in the FL PSD-95 protein influences the *in vitro* binding affinities of our trimeric ligands. We therefore examined the trimeric ligands (**12–17** and **21**) for their affinity to FL PSD-95 in the FP assay, which showed that the respective affinities of the trimeric ligands (**12–17** and **21)** as well as the dimeric ligand **20** for FL PSD-95 were similar to those determined for PSD-95 PDZ1-2-3 (Table 1). The only discrepancy was seen for trimeric ligand **20** containing the very long PEG28 linker, where the affinity was approximately 6-fold lower for FL PSD-95 versus the PDZ1-2-3 construct (Table 1). This difference could be explained by the additional bulkiness of PDZ3-SH3-GK supramodule in FL PSD-95 relative to the PDZ3 alone in PDZ1-2-3, which causes sterical hindrance with the long linker. Finally, the monomeric CRIPT-derived peptides also had comparable affinities to PSD-95 PDZ3 and FL PSD-95, as indicated by the truncation study (S1 Table), and thus suggests that the *in vitro* binding affinity of peptides targeting PDZ3 are not modulated by the SH3-GK domain.

### Trivalent binding examined by stopped-flow spectrometry

**Fig 5:**
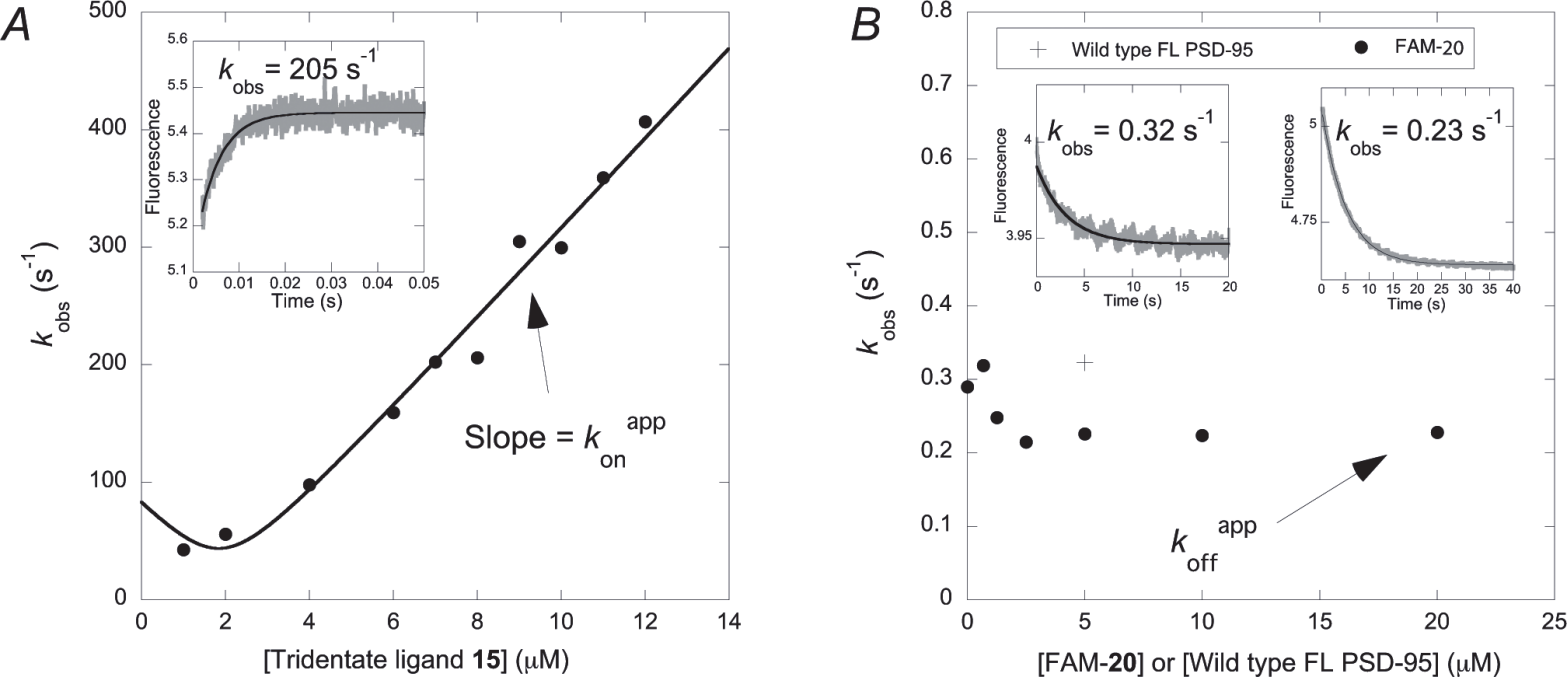
Binding kinetics for the trimeric ligand 15 with FL PSD-95. (*A*) *Inset*: FL PSD-95 I100W (2 μM) was mixed with the trimeric ligand **15** (8 μM). The observed rate constant *k*_obs_ obtained from such binding traces were plotted versus the concentration of **15**. The slope of the curve in the linear region equals the apparent association rate constant *k*_on_^app^, as indicated. (*B*) *Right inset*: A complex of FL PSD-95 (I100W) (1 μM final concentration) and trimeric ligand **15** (1 μM final concentration) was mixed with an excess of 5-FAM-labeled dimeric ligand **20** and the change in fluorescence with time was monitored. The *k*_obs_ values from the displacement experiments were plotted versus concentration of 5-FAM-**20**. At high [5-FAM-**20**], the *k*_obs_ of this displacement reaction approaches the overall dissociation rate constant *k*_off_^app^ as indicated by the arrow. *Left inset*: The complex between FL PSD-95 (I100W) and **15** was displaced by mixing with 5 μM wild type FL PSD-95. Despite a poor signal-to-noise ratio, this experiment agreed well with the displacement using 5-FAM-**20**.

To further analyze the binding of trimeric ligand **15** to FL PSD-95, we performed kinetic binding studies using stopped-flow spectrometry. Based on previous experiments with the PDZ1-2 supramodule and dimeric ligands [33,37] we introduced Trp residues in PDZ1 (I100W) and PDZ2 (I195W), respectively, in FL PSD-95. In this way we could follow the change in Trp fluorescence with time upon binding of trimeric ligands to FL PSD-95. The binding of a trimeric ligand to three different binding sites on a protein is a multi-step process, which may give rise to complex kinetics. However, the experimental results were surprisingly simple (S5 Table and Fig. 5). The association kinetics were well described by a single exponential function (Fig. 5A) and the extracted observed rate constants yielded *k*_on_^app^ values of 38 μM^-1^s^-1^ for FL PSD-95 (I100W) and 23 μM^-1^s^-1^ for FL PSD-95 (I195W). The observed *k*_on_^app^ values for multivalent interactions are sums of individual microscopic *k*_on_ values for each particular ligand-protein interaction.[37] While the two values represent two different kinetic phases, the higher one is likely the overall apparent *k*_on_ value and can be used to calculate the overall *K*_d_ as *k*_off_^app^/*k*_on_^app^.

The overall dissociation rate constant *k*_off_^app^ was determined in a displacement reaction. Here, FL PSD-95 (I100W or I195W) was pre-incubated with the trimeric ligand **15** and mixed with a competing ligand, either 5-FAM-labeled dimeric ligand **20** (5-FAM-**20**) (Fig. 5B), wild type FL PSD-95 (Fig. 5B) or a N-terminally dansylated CRIPT hexapeptide YKQTSV (dansyl-**1**) (S1 Fig). At high concentrations of the competing ligand the observed rate constant in the experiment will be equal to the overall dissociation rate constant *k*_off_^app^. The experimental trace obtained with 5-FAM-**20** was well described by a single exponential function with an observed rate constant of around 0.23 s^-1^ for both Trp mutants of FL PSD-95. Thus, the *K*_d_ of **15** can be estimated as 0.23/38 = 6.1 nM, which is in good agreement with the *K*_i_ value from the FP assay (*K*_i_ = 3.3 ± 0.7 nM, Table 1). To investigate the effect of the third ligand, we performed similar binding and displacement experiments with the dimeric ligand **20** (S5 Table). Comparison of the kinetic parameters obtained for FL PSD-95 with the trimeric ligand **15** (*k*_off_ = 0.23 s^-1^, *k*_on_ = 38 M^-1^s^-1^) and the dimeric ligand **20** (*k*_off_ = 0.68 s^-1^, *k*_on_ = 34 M^-1^s^-1^), respectively shows that inclusion of the third interaction in the trimeric ligand increases the affinity by slowing down the dissociation of the complex.

Displacement with wild type FL PSD-95 yielded noisy experimental traces due to the high Trp concentration in the experiment. Nevertheless, data agreed well with those of 5-FAM-**20** (0.32 s^-1^ at 5 μM wild type FL PSD-95, Fig. 5B). A likely model consistent with these data is that the third ligand decreases the probability of simultaneous dissociation of all three ligands in the trivalent interaction.

Finally, additional evidence for a trivalent interaction between FL PSD-95 and **15** comes from kinetic experiments with dansyl-**1** (S1 Fig). The binding kinetics for dansyl-**1** with FL PSD-95 was biphasic. The association rate constant for the linear phase was determined for all three FL PSD-95 variants to be 6 μM^-1^s^-1^ (S1B Fig, S5 Table), which is slightly lower than the one measured with a single PDZ3 domain (*k*_on_ = 10 μM^-1^s^-1^).[47] Since simultaneous binding to several PDZ domains should likely result in kinetic phases with higher apparent *k*_on_ values, this result indicates that the observed binding of dansyl-**1** is mainly to PDZ3, in agreement with FP data (S1 Table). The second phase that appears rather constant in the measured concentration interval (*k*_obs_ of 4–5 s^-1^) suggests that a conformational change occurs during the binding reaction. With this information at hand, we attempted to displace trivalent compound **15** with dansyl-**1**: At a large excess of dansyl-**1** we observed an increase in dansyl fluorescence on mixing with the FL PSD-95 (I100W)/trimeric inhibitor **15** complex (S1C Fig). The observed rate constant (1.3 s^-1^) is close to the one observed previously for dansyl-**1**/PDZ3 (1.6 s^-1^).[47] The low affinity of CRIPT towards PDZ1 and PDZ2 (97 ± 18 μM and 25 ± 1,6 μM, respectively) and the equally low affinity of IETAV for PDZ3 (>50 μM, S1 Table) suggest that dansyl-**1** exclusively competes with **15** for binding to PDZ3. It is also likely that dansyl-**1** can compete out the PDZ3 binding moiety of **15** without complete dissociation of **15** from FL PSD-95. Thus, this experiment corroborates the trivalent interaction between compound **15** and FL PSD-95.

## Conclusion

The MAGUK proteins are scaffolding proteins with numerous important functions in the CNS, where in particular PSD-95 has shown promise as a therapeutic target for a range of diseases. Recently, it was shown that MAGUK proteins have an architecture in which the PDZ1-2 and PDZ3-SH3-GK domains, respectively, form two independent supramodules. However, the functional consequence of this organization is largely unknown. Here we have explored the concept of trimeric ligands targeting MAGUK proteins as a way to increase affinity towards these proteins and potentially develop useful pharmacological tools. The trimeric ligands are supposed to simultaneously target the three PDZ domains (PDZ1, PDZ2 and PDZ3 of the MAGUKs) and in this way span the linker between the two supramodules. First, we demonstrated that a trimeric ligand (**17**) containing a very long PEG28 linker had a affinity than a dimeric ligand (**11)** to PDZ1-2-3 of PSD-95, representing a proof of concept for trivalent binding to PSD-95. Further optimization identified a trimeric ligand **15**, containing a PEG12 linker, which was found to have optimal length for targeting the PDZ1-2-3 proteins of PSD-95 and showed an 8-fold improvement in affinity compared with our previously published high affinity dimeric inhibitor (**20**),[33,34] with a *K*_i_ value of 3.3 nM. Thus, these ligands are the most potent inhibitors of MAGUK PDZ domains described. When examining the trimeric ligands at PDZ1-2-3 of PSD-93, SAP-97 and SAP-102, we generally found that affinities were similar to those of PSD-95. This indicated that the sequence differences of the peptide region linking the PDZ1-2 and PDZ3-SH3-GK supramodules of the four MAGUK proteins does not lead to differences with respect to interacting with trimeric ligands. A number of binding experiments, including a trimeric ligand with disrupted PDZ3 affinity, indicated that the very high affinities of the trimeric ligands, **15** in particular, was caused by a true trivalent interaction with PDZ1-2-3 of the MAGUK proteins. We also found that generally, PSD-95 PDZ1-2-3 and FL PSD-95 did not differ in their respective affinities towards the monomeric, dimeric and trimeric ligands, suggesting that the SH3/GK domains do not significantly modulate PDZ ligand binding of PSD-95 *in vitro*. Finally, kinetic binding experiments were employed showing that the increased affinity of trimeric ligands is likely due to a decrease in the probability of simultaneous dissociation of all three binding moieties in the trivalent interaction.

## Supporting Information

S1 Text Experimental section. (PDF)

S1 Table Affinity of truncated CRIPT C-terminal peptides toward PSD-95 PDZ3 and PSD-95 FL as determined by FP^*a*^. (PDF)

S2 Table Affinity toward the PDZ domains of PSD-95 of 1, 17 and 19 as determined by FP^*a*^. (PDF)

S3 Table IC_50_ values in nM of tridentate ligands and dimeric ligand 20 toward the PDZ1-2-3 domains of PSD-93, PSD-95, SAP-97 and SAP-102 as well as FL PSD-95 as determined by FP. (PDF)

S4 Table Control experiments for the expressed MAGUK PDZ1-2-3 proteins and FL PSD-95^*a*^. (PDF)

S5 Table Kinetic parameters for the interaction between FL PSD-95 with an engineered Trp in either PDZ1 (I100W) or PDZ2 (I195W) and tridentate ligand 15 and dansyl-1. (PDF)

S6 Table Characterization of tridentate ligands. (PDF)

S7 Table Characterization of building blocks. (PDF)

S1 Fig Binding kinetics for dansyl-CRIPT-06 to FL PSD-95 I100W. (PDF)
