# Supplementary material for: Targeting Protein-Protein Interactions with Trimeric Ligands: High Affinity Inhibitors of the MAGUK Protein Family
Source: PLoS One. 2015 Feb 6;10(2):e0117668. doi: 10.1371/journal.pone.0117668 (PMC4319893; doi:10.1371/journal.pone.0117668)
Supplement: S1 Text — (PDF) [file pone.0117668.s001.pdf]

Experimental section

Protein expression and validation of function

Full-Length PSD-95 was expressed using the pET32m3C expression vector using a previously reported protocol.[1] Synthetic DNA encoding the human MAGUK PDZ1-2-3 proteins (supplied by Geneart, Life Technologies, Lohne, Germany) was cloned into a modified pRSET expression vector (Invitrogen, Carlsbad, CA), containing a 7xHis-tag for purification. The following residues were included in the constructs: PSD95-PDZ1-2-3: 61-401 (numbering refers to the residues in the parental human full length PSD-95α withouth exon 4b, as used by others[2-4]), PSD-95 FL: 61-end, PSD93 PDZ1-2-3: 94-509, SAP97 PDZ1-2-3: 220-554, SAP102 PDZ1-2-3: 127-475. The constructs were expressed in BL21(DE3)pLysS competent cells (Invitrogen, Carlsbad, CA) using regular Luria-Bertani Broth (LB) medium and induction with 1mM isopropyl β-D-1-thiogalactopyranoside (IPTG) at an optic density (OD) of approximately 0.7-0.8 followed by incubated overnight at 25° C. After harvest and cell lysis (using a french press), the proteins were purified by immobilized metal-ion affinity chromatography using a His-Trap (GE life-sciences) on a Äkta FPLC system. Secondary purification was performed using a MonoQ HR 5/5 ion-exchange column. The purity of the proteins was confirmed using SDS-PAGE. To validate the integrity and function of the expressed proteins, a number of control experiments were carried out using FP assay. First, it was tested whether the proteins were able to bind the probe in a saturation binding experiment, and the K_D was calculated. Next, it was tested whether the binding was reversible by competition with the unlabeled probe, and the K_i was calculated as

previously described[5]. If $K_D \approx K_i$ then it was judged that the protein was functional (Table S4). PSD-95 PDZ1-2 was expressed as previously described.[6]

Mutagenesis

Mutagenesis was performed using synthetic primers and the standard protocol of the QuickChange Site-Directed Mutagenesis-Kit (Agilent Technologies).

Circular dichroism

CD spectra were recorded using an Olis DSM 100 CD spectrophotometer (Olis Inc., Bogart, GA, USA) in 1 mm quartz cuvettes (Starna Scientific Ltd., Essex, England). A protein concentration of 8 μM in a 50 mM Phosphate buffer, pH 7.4 was used.

Concentration determination

The concentrations of all proteins and peptides used for FP were determined by amino acid analysis (Alphalyze, Odense, Denmark). This analysis also served as a secondary confirmation of the peptide and protein identity.

References

1. Long JF, Tochio H, Wang P, Fan JS, Sala C, et al. (2003) Supramodular structure and synergistic target binding of the N-terminal tandem PDZ domains of PSD-95. J Mol Biol 327: 203-214.
2. Chi CN, Haq SR, Rinaldo S, Dogan J, Cutruzzola F, et al. (2012) Interactions outside the boundaries of the canonical binding groove of a PDZ domain influence ligand binding. Biochemistry 51: 8971-8979.

3. Doyle DA, Lee A, Lewis J, Kim E, Sheng M, et al. (1996) Crystal structures of a complexed and peptide-free membrane protein-binding domain: molecular basis of peptide recognition by PDZ. Cell 85: 1067-1076.
4. Tochio H, Hung F, Li M, Bredt DS, Zhang M (2000) Solution structure and backbone dynamics of the second PDZ domain of postsynaptic density-95. J Mol Biol 295: 225-237.
5. Nikolovska-Coleska Z, Wang R, Fang X, Pan H, Tomita Y, et al. (2004) Development and optimization of a binding assay for the XIAP BIR3 domain using fluorescence polarization. Anal Biochem 332: 261-273.
6. Bach A, Clausen BH, Møller M, Vestergaard B, Chi CN, et al. (2012) A high-affinity, dimeric inhibitor of PSD-95 bivalently interacts with PDZ1-2 and protects against ischemic brain damage. Proc Natl Acad Sci U S A 109: 3317-3322.
