# Supplementary material for: Targeting Protein-Protein Interactions with Trimeric Ligands: High Affinity Inhibitors of the MAGUK Protein Family
Source: PLoS One. 2015 Feb 6;10(2):e0117668. doi: 10.1371/journal.pone.0117668 (PMC4319893; doi:10.1371/journal.pone.0117668)
Supplement: S1 Table — (PDF) [file pone.0117668.s002.pdf]

Table S1. Affinity of truncated CRIPT C-terminal peptides toward PSD-95 PDZ3 and PSD-95 FL as determined by FP[a]

Name	Peptide sequence	K_i, PSD-95 PDZ3 (µM)	K_i, PSD-95 FL (µM)
CRIPT-11	LDTKNYKQTSV	3.0 ± 0.6	4.6 ± 0.3
CRIPT-09	TKNYKQTSV	1.5 ± 1.0	3.1 ± 0.1
CRIPT-08	KNYKQTSV	0.96 ± 0.5	2.1 ± 0.2
CRIPT-07	NYKQTSV	4.0 ± 0.3	3.7 ± 0.3
CRIPT-06 (**1**)	YKQTSV	3.2 ± 1.0	1.5 ± 0.3
CRIPT-05	KQTSV	8.2 ± 1.0[b]	24 ± 4[b]
CRIPT-2Ala (**9**)	TKNYKQASA	>50	N.D.

[a]K_D values are shown as mean ± SEM in µM, n≥3. [b]Significantly different from CRIPT-11, p<0,05, one-sided t-test. N.D.=not determined.
