# Supplementary material for: Targeting Protein-Protein Interactions with Trimeric Ligands: High Affinity Inhibitors of the MAGUK Protein Family
Source: PLoS One. 2015 Feb 6;10(2):e0117668. doi: 10.1371/journal.pone.0117668 (PMC4319893; doi:10.1371/journal.pone.0117668)
Supplement: S2 Table — (PDF) [file pone.0117668.s003.pdf]

Table S2. Affinity toward the PDZ domains of PSD-95 of **1**, **17** and **19** as determined by FP[a]

**Compound**		**PDZ1** [b]	**PDZ2** [b]	**PDZ3** [c]	**PDZ1-2** [b]	**PDZ1-2-3**
**1**	K_i (μM)	86 ± 21	10 ± 0.7	3.2 ± 0.1	15 ± 0.7	2.7 ± 0.1 [c]
**17**	K_i (μM)	3.6 ± 0.5	1.0 ± 0.1	4.1 ± 0.1	#	#
**19**	K_i (μM)	6.2 ± 0.3	1.5 ± 0.3	N.A.	1.7 ± 0.1	1.8 ± 0.2 [b]

[a]Data shown as mean ± SEM in μM, n≥3. K_i-values calculated according to Nikolovska-Coloska et al.,[1]. N.A.= No affinity, #= calculated K_i is negative; [b]Monomeric GluN2B used as probe; [c]Monomeric CRIPT used as probe.
