# Supplementary material for: Targeting Protein-Protein Interactions with Trimeric Ligands: High Affinity Inhibitors of the MAGUK Protein Family
Source: PLoS One. 2015 Feb 6;10(2):e0117668. doi: 10.1371/journal.pone.0117668 (PMC4319893; doi:10.1371/journal.pone.0117668)
Supplement: S3 Table — (PDF) [file pone.0117668.s004.pdf]

Table S3. IC_{50} values in nM of tridentate ligands and dimeric ligand **20** toward the PDZ1-2-3 domains of PSD-93, PSD-95, SAP-97 and SAP-102 as well as FL PSD-95 as determined by FP

Ligand	PSD-93 PDZ1-2-3	PSD-95 PDZ1-2-3	SAP-97 PDZ1-2-3	SAP-102 PDZ1-2-3	PSD-95 FL
**12**	28 ± 2.7	31 ± 3.6	21 ± 3.0	22 ± 3.8	31 ± 2.3
**13**	27 ± 4.5	22 ± 3.9	23 ± 3.1	25 ± 3.4	30 ± 2.2
**14**	21 ± 6.4	38 ± 5.9	21 ± 6.2	23 ± 7.4	40 ± 6.6
**15**	9.8 ± 0.9	13 ± 1.4	8.3 ± 1.2	8.6 ± 1.3	13 ± 1.3
**16**	22 ± 4.1	31 ± 2.5	24 ± 4.6	24 ± 1.6	37 ± 2.9
**27**	14 ± 1.4	25 ± 2.3	20 ± 0.9	28 ± 2.5	76 ± 7.0
**21**	36 ± 3.9	82 ± 6.6	31 ± 4.0	36 ± 5.8	86 ± 12.5
**20**	36 ± 1.8	58 ± 3.0	29 ± 2.2	68 ± 10.3	65 ± 4.5

[a] Data shown as mean ± SEM in nM, n≥3.
