# Supplementary material for: Targeting Protein-Protein Interactions with Trimeric Ligands: High Affinity Inhibitors of the MAGUK Protein Family
Source: PLoS One. 2015 Feb 6;10(2):e0117668. doi: 10.1371/journal.pone.0117668 (PMC4319893; doi:10.1371/journal.pone.0117668)
Supplement: S4 Table — (PDF) [file pone.0117668.s005.pdf]

Table S4. Control experiments for the expressed MAGUK PDZ1-2-3 proteins and FL PSD-95[a].

Probe		PSD-93 PDZ1-2-3	PSD-95 PDZ1-2-3	SAP-97 PDZ1-2-3	SAP-102 PDZ1-2-3	PSD-95 FL
**GluN2B**	K_d	1.3 ± 0.1	1.9 ± 0.1	1.1 ± 0.1	2.5 ± 0.1	1.4 ± 0.1
	K_i	2.2 ± 0.1	6.6 ± 0.3	1.4 ± 0.1	1.1 ± 0.2	4.6 ± 0.3
**1**	K_d	0.83 ± 0.02	2.1 ± 0.1	1.6 ± 0.1	0.59 ± 0.05	1.2 ± 0.1
	K_i	0.93 ± 0.02	2.7 ± 0.1	1.0 ± 0.1	0.26 ± 0.04	4.9 ± 0.4
**18**	K_d	0.0044 ± 0.0001	0.011 ± 0.001	0.0074 ± 0.0001	0.011 ± 0.001	0.011 ± 0.001
	K_i	0.0019 ± 0.0003	0.015 ± 0.001	0.0060 ± 0.0005	0.012 ± 0.001	0.016 ± 0.001

[a] Data shown as mean ± SEM in μM, n≥3. K_i values calculated according to Nikolovska-Coloska et al., 2004.[1]
