# Supplementary material for: Targeting Protein-Protein Interactions with Trimeric Ligands: High Affinity Inhibitors of the MAGUK Protein Family
Source: PLoS One. 2015 Feb 6;10(2):e0117668. doi: 10.1371/journal.pone.0117668 (PMC4319893; doi:10.1371/journal.pone.0117668)
Supplement: S5 Table — (PDF) [file pone.0117668.s006.pdf]

Table S5. Kinetic parameters for the interaction between FL PSD-95 with an engineered Trp in either PDZ1 (I100W) or PDZ2 (I195W) and tridentate ligand **15** and dansyl-**1**.

**Ligand**	**PSD-95 FL I100W**		**PSD-95 FL I195W**		**PSD-95 wild-type**	
	k_{on}^{app} ($\mu M^{-1}s^{-1}$)	k_{off}^{app} (s^{-1})	k_{on}^{app} ($\mu M^{-1}s^{-1}$)	k_{off}^{app} (s^{-1})	k_{on}^{app} ($\mu M^{-1}s^{-1}$)	k_{off}^{app} (s^{-1})
**15**	38 ± 1.3	0.23 ± 0.01	23 ± 1.1	0.19 ± 0.02	ND	ND
**20**	34 ± 4.5	0.68 ± 0.05	15 ± 2.6	ND	ND	ND
**1**	6.3 ± 0.19	ND	6.3 ± 0.13	ND	6.0 ± 0.1	ND

[a] ND, not determined
