# Supplementary material for: Targeting Protein-Protein Interactions with Trimeric Ligands: High Affinity Inhibitors of the MAGUK Protein Family
Source: PLoS One. 2015 Feb 6;10(2):e0117668. doi: 10.1371/journal.pone.0117668 (PMC4319893; doi:10.1371/journal.pone.0117668)
Supplement: S6 Table — (PDF) [file pone.0117668.s007.pdf]

Table S6. Characterization of tridentate ligands

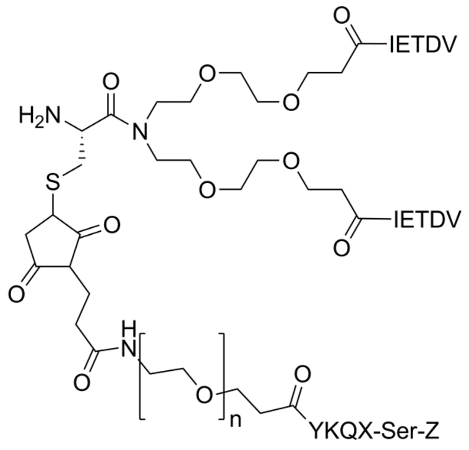

Compd.	n	X	Z	Yield	purity	formula	Calc. Mass [M+H$^+$]	Obs. Mass LC-MS (m/z)	Obs. Mass MALDI-TOF (m/z)
**12**	4	T	V	1.1 mg (12%)	>95%	$C_{115}H_{188}N_{22}O_{48}S$	2678.27	893.7, 1340.0	2679.34
**13**	8	T	V	2.0 mg (22%)	>95%	$C_{123}H_{204}N_{22}O_{52}S$	2853.37	952.2, 1427.7	2877.34 (+Na$^+$)
**14**	10	T	V	38.9 mg (61%)	>95%	$C_{127}H_{212}N_{22}O_{54}S$	2942.42	736.7, 981.9, 1472.3	2943.19
**15**	12	T	V	71.8 mg (81%)	>95%	$C_{131}H_{220}N_{22}O_{56}S$	3030.48	1011.3, 1516.2	3031.60
**16**	16	T	V	0.6 mg (6%)	>95%	$C_{139}H_{236}N_{22}O_{60}S$	3206.58	802.3, 1069.7, 1603.9	3208.94
**17**	28	T	V	1.8 mg (38%)	>95%	$C_{163}H_{284}N_{22}O_{72}S$	3734.90	747.7, 934.7, 1245.7	3773.87 (+K$^+$)
**21**	12	A	A	5.0 mg (8%)	>95%	$C_{128}H_{214}N_{22}O_{55}S$	2972.43	744.2, 992.0, 1487.0	2973.94
