# Supplementary material for: Targeting Protein-Protein Interactions with Trimeric Ligands: High Affinity Inhibitors of the MAGUK Protein Family
Source: PLoS One. 2015 Feb 6;10(2):e0117668. doi: 10.1371/journal.pone.0117668 (PMC4319893; doi:10.1371/journal.pone.0117668)
Supplement: S7 Table — (PDF) [file pone.0117668.s008.pdf]

Table S7. Characterization of building blocks

Compd.	Yield	purity	formula	Calc. Mass [M+H$^+$]	Obs. Mass ESI (m/z)
**1**	0.62 g (100%)	>95%	$C_{68}H_{98}N_8O_{13}$	1235.73	893.1 (-Boc,-Trt), 993.0 (-Trt), 1235.7
**2**	25.8 mg (23%)	>95%	$C_{50}H_{78}N_{10}O_{19}$	1123.54	562.5, 1123.4
**3**	28.7 mg (30%)	>95%	$C_{58}H_{94}N_{10}O_{23}$	1299.65	650.6, 1299.6
**4**	29.9 mg (43%)	>95%	$C_{62}H_{102}N_{10}O_{25}$	1387.70	463.4, 694.5, 1387.7
**5**	43.2 mg (43%)	>95%	$C_{66}H_{110}N_{10}O_{27}$	1475.75	492.5, 738.5, 1475.7
**6**	2.9 mg (2%)	>95%	$C_{74}H_{126}N_{10}O_{31}$	1651.86	551.3, 826.5
**7**	5.6 mg (4.2%)	>95%	$C_{98}H_{174}N_{10}O_{43}$	2180.18	545.8, 727.6, 1090.7
**8**	112 mg (100%)	>95%	$C_{63}H_{104}N_{10}O_{26}$	1417.71	473.4, 709.5, 1417.7
**9**	139 mg (50%)	>95%	$C_{61}H_{84}N_8O_{12}$	1120.62	879.0 (-Trt), 1120.4
**11**	1.34 g (43%)	>95%	$C_{65}H_{110}N_{12}O_{29}S$	1555.72	787.4, 1555.7
