# Supplementary material for: Targeting Protein-Protein Interactions with Trimeric Ligands: High Affinity Inhibitors of the MAGUK Protein Family
Source: PLoS One. 2015 Feb 6;10(2):e0117668. doi: 10.1371/journal.pone.0117668 (PMC4319893; doi:10.1371/journal.pone.0117668)
Supplement: S1 Fig — (PDF) [file pone.0117668.s009.pdf]

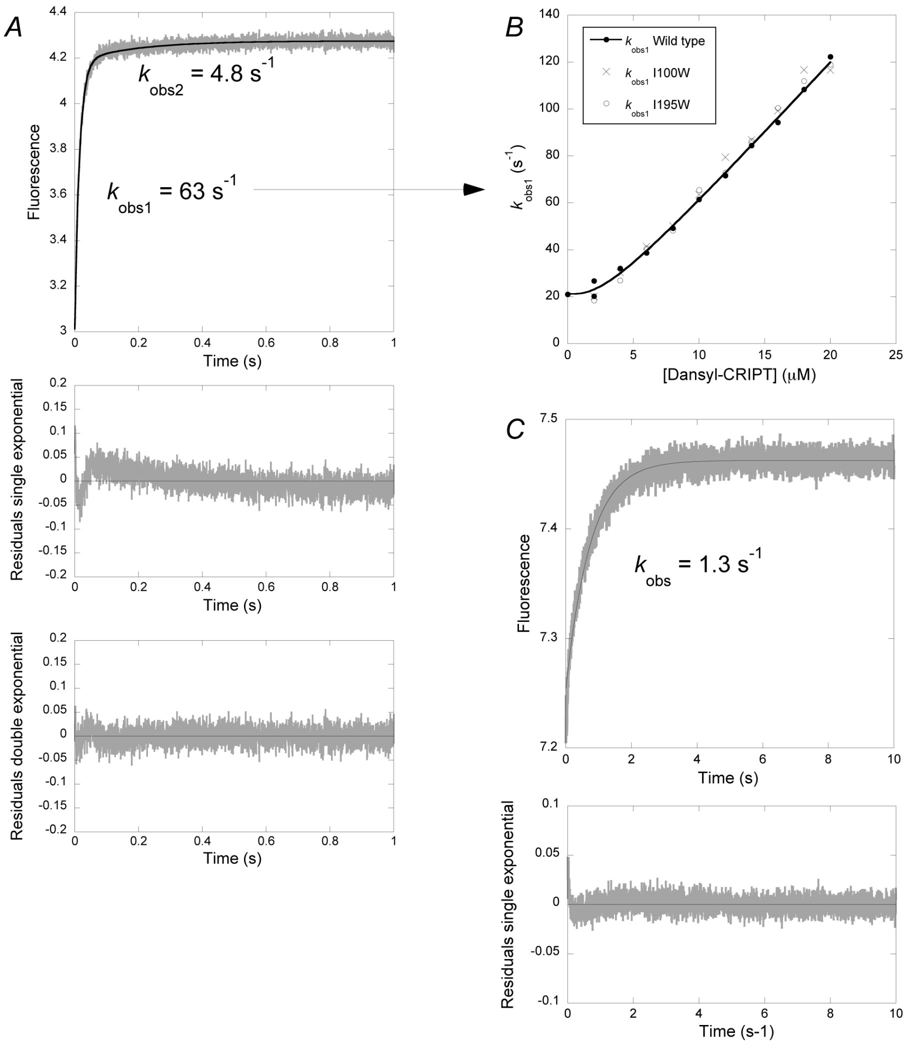

Figure S1. Binding kinetics for dansyl-**1** to FL PSD-95 I100W. (*A*) Experimental binding trace resulting from mixing 2 μM FL PSD-95 (I100W) with 10 μM dansyl-**1**. The trace was fitted to a double exponential equation to obtain two observed rate constants, k_{obs1} and k_{obs2}, respectively. The rate constant k_{obs1} associated with the fast phase was plotted versus concentration of dansyl-**1** (panel *B*) to obtain the association rate constant k_{on} (Table S5) by fitting to an equation describing association of two molecules.[1] The slow phase described by k_{obs2} was constant (~4 s^{-1}) in the measured concentration interval (2-20 μM dansyl-**1**) and might be related to a conformational change in PSD-95. (*C*) Dissociation of a complex between PSD-95 (I100W) and tridentate inhibitor **15** by 100 μM dansyl-**1** monitored by excitation at 330 nm and emission at >420 nm (i.e., change in dansyl fluorescence). Fit to a single exponential function yielded a kobs of 1.3 s^{-1}. While this experimental trace was satisfactorily described by a single exponential, the kinetics as monitored by Trp fluorescence (excitation at 280 nm and emission around 330 nm, not shown) were biphasic with a fast phase (~2 s^{-1}) that could be related to the apparent dissociation rate constant of the CRIPT hexapeptide moiety of tridentate inhibitor **15** and a slower phase (~0.4 s^{-1}) possibly reflecting complete dissociation of **15** before binding of dansyl-**1**.
